# Site of Allergic Airway Narrowing and the Influence of Exogenous Surfactant in the Brown Norway Rat

**DOI:** 10.1371/journal.pone.0029381

**Published:** 2012-01-19

**Authors:** Sana Siddiqui, Kimitake Tsuchiya, Paul-André Risse, Sharon R. Bullimore, Andrea Benedetti, James G. Martin

**Affiliations:** Meakins-Christie Laboratories, Department of Medicine, McGill University, and the Research Institute of the McGill University Health Centre, Montréal, Québec, Canada; University Hospital Freiburg, Germany

## Abstract

**Background:**

The parameters R_N_ (Newtonian resistance), G (tissue damping), and H (tissue elastance) of the constant phase model of respiratory mechanics provide information concerning the site of altered mechanical properties of the lung. The aims of this study were to compare the site of allergic airway narrowing implied from respiratory mechanics to a direct assessment by morphometry and to evaluate the effects of exogenous surfactant administration on the site and magnitude of airway narrowing.

**Methods:**

We induced airway narrowing by ovalbumin sensitization and challenge and we tested the effects of a natural surfactant lacking surfactant proteins A and D (Infasurf®) on airway responses. Sensitized, mechanically ventilated Brown Norway rats underwent an aerosol challenge with 5% ovalbumin or vehicle. Other animals received nebulized surfactant prior to challenge. Three or 20 minutes after ovalbumin challenge, airway luminal areas were assessed on snap-frozen lungs by morphometry.

**Results:**

At 3 minutes, R_N_ and G detected large airway narrowing whereas at 20 minutes G and H detected small airway narrowing. Surfactant inhibited R_N_ at the peak of the early allergic response and ovalbumin-induced increase in bronchoalveolar lavage fluid cysteinyl leukotrienes and amphiregulin but not IgE-induced mast cell activation *in vitro*.

**Conclusion:**

Allergen challenge triggers the rapid onset of large airway narrowing, detected by R_N_ and G, and subsequent peripheral airway narrowing detected by G and H. Surfactant inhibits airway narrowing and reduces mast cell-derived mediators.

## Introduction

The constant phase model of respiratory mechanics has been proposed for the partitioning of pulmonary responses to the airways and tissues [1], [2]. Changes in mechanics directly attributable to narrowing of the conducting airways is detected by R_N_ and changes in tissue viscous resistance (tissue viscance or tissue damping) are detected by the G parameter [1]. Tissue damping increases with heterogeneity of bronchoconstriction and hyperinflation [3], [4]. Hyperinflation and inhomogeneity of ventilation distribution also contribute to increases in tissue elastance (H parameter) [5]. Since airway resistance is predominantly contributed by the larger conducting airways, the constant phase model parameters may partition the airway responses into those related to the large airways (R_N_) and to the peripheral lung (G and H) [2]. The site of airway narrowing following methacholine or allergen-induced bronchoconstriction may be directly assessed by snap-freezing lungs and performing morphometric measurements [6].

Allergen challenge of sensitized subjects may result in early and late responses [1]. The early response occurs within minutes after allergen exposure and results from mast cell degranulation and the release of preformed biogenic amines including histamine and serotonin and the *de novo* synthesis of cysteinyl leukotrienes (CysLTs) [7], [8]. These mediators also cause a vascular leak into the airways [9], [10]. Microvascular leak of proteins as well as surfactant phospholipid changes such as that occurring during allergen-induced airway reactions may disrupt surfactant function [11], [12] and serve as an additional mechanism for airway narrowing following allergen challenge. Surfactant administration may be beneficial in restoring surfactant activity [13] and we reasoned it would preferentially reduce peripheral airway narrowing following allergen challenge. A previous study of allergen challenge of the sensitized rat by the inhalational route has shown that alterations in respiratory mechanics reflect a mixed large airway and peripheral lung response [14], suggesting that this model was suited to addressing the effects of surfactant.

The first objective of the current study was to compare the site of airway narrowing as determined by the constant phase model and by morphometry during the early allergic response (EAR). The second objective was to assess the potential modulation of changes in airway mechanics following Ova or serotonin-induced bronchoconstriction by the administration of surfactant. To examine if surfactant might inhibit the allergic response itself, we also assessed the mast cell mediators, CysLTs and amphiregulin, in bronchoalveolar lavage fluid immediately after the peak of the EAR after Ova challenge. Additionally we examined the effects of surfactant on Ova-triggered mast cell activation *ex vivo*.

## Methods

### Ethics Statement

The study protocol was approved by the Animal Care Committee of McGill University.

### Animals and sensitization to ovalbumin

Male Brown Norway (BN) rats, 6–8 weeks old and weighing 160–180 g (SsN substrain) (Harlan/UK), were sensitized subcutaneously with 1 mg ovalbumin (Ova) and 100 mg aluminum hydroxide dissolved in 1 mL of phosphate buffered saline (PBS) [15]. Concurrently, the rats were injected intraperitoneally with 2×10^9^ *Bordetella pertussis* heat-killed bacteria, an adjuvant (provided by T. Issekutz, Dalhousie, University, Halifax, NS, Canada) [15].

### Measurement of early allergic response (EAR) to ovalbumin challenge

Fourteen days after sensitization, rats were endotracheally intubated under light pentobarbital anesthesia and then administered an aerosol challenge for 1 minute, with either 5% Ova or the PBS control, using an ultrasonic nebulizer. Paralysis was induced with 1 mg/kg pancuronium bromide intraperitoneally (Sandoz Canada Inc, QC, Canada). The animals were placed on a heating pad and their body temperature was monitored with a rectal thermometer. The animals were ventilated at a tidal volume of 8 mL/kg, a breathing frequency of 90 breaths/minute and an end-expiratory pressure of 2.5 cmH_2_O using a computer controlled small animal ventilator ((Flexivent, Scireq, Montréal, QC, Canada) up to 20 minutes after the Ova or PBS challenge. Respiratory system mechanics were assessed every 15 seconds using the constant phase model [16]. The latter model fits the data to an equation that has four parameters and these are estimated by solving the following:
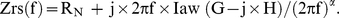
where Z is input impedance and expresses the combined effects of resistance, compliance and inertance as a function of frequency, R_N_ is Newtonian resistance (frequency independent), Iaw is airway inertance and is dominated by the mass of gas in the central airways, and impedance of tissues is accounted for by both G and H. G (tissue damping) is closely related to peripheral airway and tissue resistance and reflects energy dissipation in the lung tissues, j is an imaginary number, H is tissue elastance and reflects energy storage in the tissues, α is 2/πtan^−1^(H/G) and f is respiratory frequency.

### Effects of surfactant on respiratory mechanical responses to ovalbumin challenge

Sensitized rats were anesthetized, paralyzed and mechanically ventilated as described above. Following a baseline recording of mechanics, the animals were administered an ultrasonic nebulization of 200 µL of a commercially available surfactant (∼7 mg phospholipids), Infasurf® (ONY, Inc, Amherst, NY) or saline, for 5 minutes. This intervention was immediately followed by an Ova or PBS challenge for 1 minute. The animals had measurements of respiratory mechanics every 15 seconds for the subsequent 20 minutes. Infasurf® contains natural phospholipids, neutral lipids and hydrophobic surfactant associated proteins in 0.9% sodium chloride including surfactant protein (SP)-B [17], [18]. According to the manufacturer, Infasurf® does not contain surfactant proteins A and D, collectins that have been shown to have immunomodulatory effects (as reviewed in [19]–[21]).

### Assessment of airway narrowing by morphometry

Separate groups of animals underwent sensitization and challenge with Ova as described above but were sacrificed ∼3 minutes or at 20 minutes following challenge and the lungs were snap-frozen for morphometric assessment (n = 4−6). A constant pressure of 4 cmH_2_O was applied during freezing so as to fix the lungs at a lung volume within the range of tidal breathing during fixation [6], [22]. The chest cavity was rapidly opened and liquid nitrogen was poured covering the lungs [6]. The lungs were removed and immersed in Carnoy's solution and stored at −80°C overnight [6]. The following day the lungs were processed in a series of modified Carnoy's solutions and the tissue was paraffin-embedded [6].

Histological sections (5 µm) were cut from mid-sagittal and para-hilar regions of the lung and were stained with hematoxylin and eosin. The lumen area was traced for at least 10 airways per animal and the ideal lumen area was calculated from the internal basement perimeter (P_BM_) on the assumption that the fully dilated airway is a circle. All airways in the whole lung section whose aspect ratio (maximal to minimal internal diameter) was less than 2, were included in the analyses. The median P_BM_ of the airways studied after a single challenge was 0.89 mm. To assess airway narrowing heterogeneity, the coefficient of variation of the airway lumina was calculated for airways >0.89 mm and for those airways <0.89 mm. Lung sections from a separate group of Ova-sensitized animals were stained with toluidine blue for the assessment of mast cell distribution (n = 8) and smooth muscle (SM) α-actin for airway smooth muscle (ASM) assessment (n = 6).

### Assessment of cysteinyl leukotriene and amphiregulin concentrations in the bronchoalveolar lavage (BAL) fluid at the peaks of EAR

Separate groups of animals were used to assess the CysLTs (n = 5−8/group) and amphiregulin concentrations (n = 7−9/group) in the BAL fluid following Ova or PBS challenge. Two weeks after sensitization with Ova, the animals were anaesthetized, paralyzed and mechanically ventilated as described above. Saline or surfactant was administered for 5 minutes using an ultrasonic nebulizer followed by either an Ova or PBS challenge. At peak EAR, the animals were sacrificed and underwent BAL. BAL was performed using sterile 1× PBS, which was administered into the lungs and then drawn out with a syringe. The first 5 mL of BAL fluid was stored at −80°C for further analysis. Amphiregulin levels were assayed using the human Amphiregulin kit (R&D Systems, Minneopolis, MN). The lower limit of detection for amphiregulin is 15.6 pg/mL. For the CysLT measurement, samples were extracted using Sep-Pak cartridges (Waters, Milford, MA) and subsequently analyzed after 8-fold dilution using the CysLT Express EIA Kit (Cayman Chemical Company, MI, USA). The CysLT EIA kit typically has a detection limit (80% B/Bo) of 20 pg/mL.

### Assessment of serotonin-induced bronchoconstriction in the presence of surfactant

Serotonin (serotonin hydrochloride, 5-HT, Sigma-Aldrich Canada Ltd., Oakville, ON, Canada) was used to induce the bronchoconstriction. 5-HT (30 mg/mL) was nebulized to non-sensitized, anesthetized, and paralyzed BN rats for 20 seconds (n = 8) using the small animal ventilator (Flexivent) [9]. Another group of animals (n = 8) was administered nebulized surfactant (Infasurf®, ONY, Inc., NY, US) for 5 minutes prior to the 5-HT administration. Subsequently, the bronchoconstrictive response was assessed using the constant phase model over 20 minutes.

### Assessment of intracellular calcium release in RBL-2H3 mast cell line

The RBL (rat basophilic leukemia)-2H3 mast cell line (American Type Culture Collection, USA) was maintained in Dulbecco's modified Eagle medium (DMEM) with 20% fetal bovine serum and antibiotics (penicillin-streptomycin-amphotericin B) Cells were plated on glass coverslips until they reached confluence. About 4 hours prior to measurements, serum was removed and replaced by PIPES buffer containing 140 mM NaCl, 5 mM KCl, 0.6 mM MgCl2, 1.0 mM CaCl2, 5.5 mM glucose and 10 mM piperazine-N-N′-bis (2-ethanesulfonic acid); pH 7.4). For the experiments performed in the absence of external calcium, PIPES–EGTA buffer (1.0 mM EGTA) was used. Thirty minutes prior to each measurement, RBL-2H3 cells were loaded with 5 µM Fura-2 AM (Molecular Probes, Eugene, OR), mounted in a Leiden chamber (Medical Systems, Greenville, NY) and viewed under the 40× oil immersion objective on an inverted microscope (Olympus, Tokyo, Japan). Five minutes prior to measurements, 10% of either control (saline) or natural exogenous surfactant was added to the medium. Calcium (Ca^2+^) signaling was triggered by addition of Ova and serum from Ova-sensitized and challenged rat solution (final concentration: 50 µg/ml Ova, 5% serum) premixed 30 minutes prior to the challenge. Fura-2 was alternatively excited at 340 nm and 380 nm with a PTI Deltascan 1 dual monochromator illuminator (Photon Technology International, Princeton, NJ). Additionally, fluorescent properties of the FURA-2 were verified *in vitro* by performing excitation-scan fluorescence measurements in presence of 0 and 39 µM Ca^2+^ with surfactant or saline. Ratios (340/380) were calculated. Rmax and Rmin were calculated in the cell line in response to ionomycin (10 µM) in PIPES and PIPES- EGTA buffer respectively. Fluorescent ratios were converted into Ca^2+^ concentration using the formula of Grynkiewicz (10).

### Statistical analysis

Data are presented as mean+SEM. The statistical analysis was performed using GraphPad Prism, version 5 (GraphPad Software Inc., San Diego, CA) as well as SAS v. 9.2 (Cary, NC). Paired means were compared using two-tailed t-tests. Linear regression was performed using the method of least squares. Comparison of morphometric data between the Ova and PBS-challenged animals was assessed using a linear mixed model estimated via restricted maximum likelihood with the independent variables being size, Ova and interaction between Ova and size and dependent variable being predicted luminal ratio. At 20 minutes, a term for the square of size was also included. This model adjusts for correlation between airways from the same rats by including a random intercept.

For the mechanical parameters of the total respiratory resistance of the surfactant groups, a one-way ANOVA and Newman-Keuls test was performed on data at 3 and 20 minutes. For the corresponding morphometry at 3 minutes, a one-way ANOVA and Bonferroni correction was used for comparisons of interest. A one-way ANOVA and Newman-Keuls post hoc test was performed on the data for CysLTs and amphiregulin. To assess mechanical parameters of the response in the 5-HT and surfactant + 5-HT groups, two-tailed t-tests were performed for data immediately after the 5-HT administration and at 5, 10, 15 and 20 minutes.

## Results

### Early allergic response: Constant phase parameters

**Figure 1:**
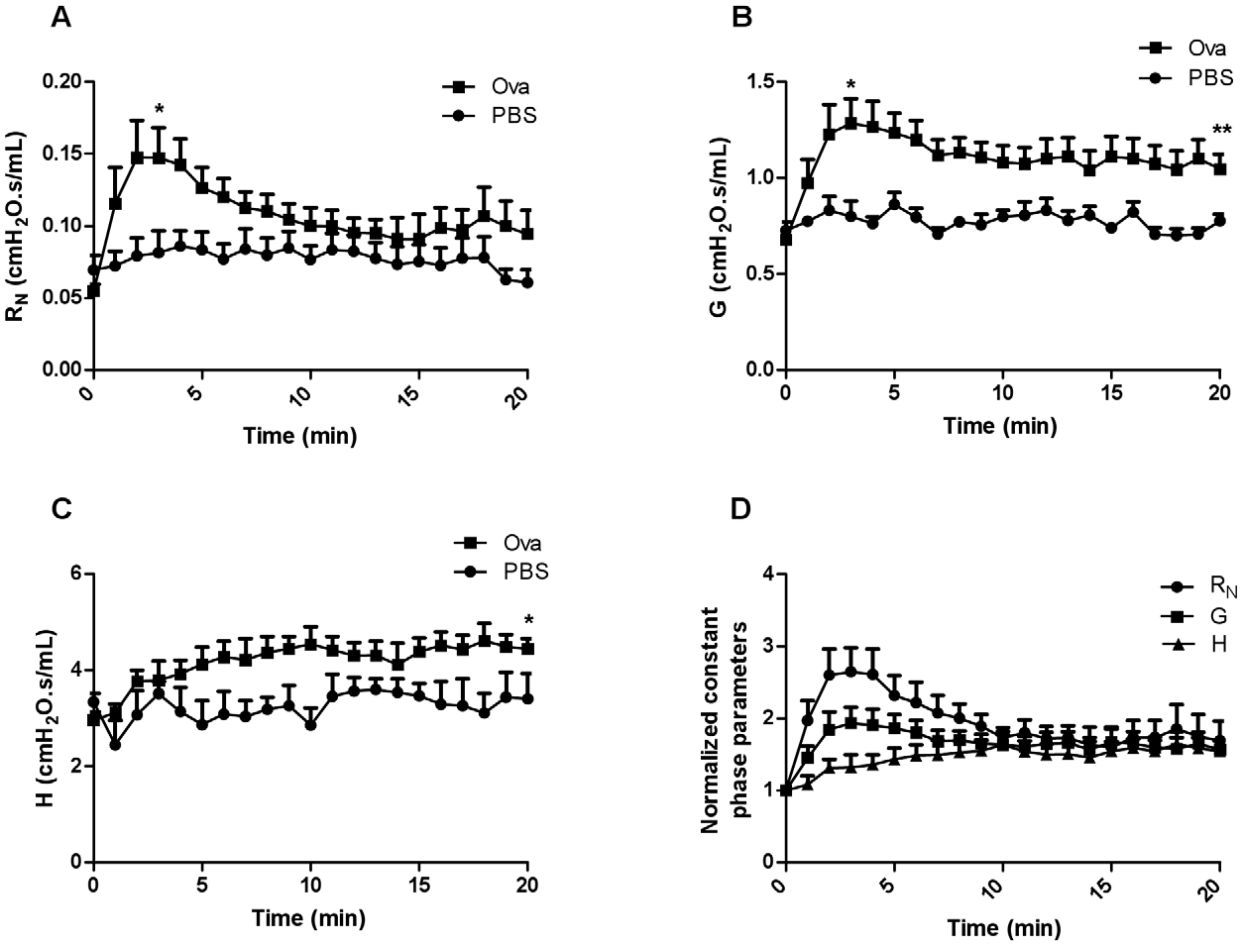
R_N_, G, and H over the 20 minute period following aerosol challenge with PBS or Ova and normalized constant phase parameters over time in Ova-challenged animals. (A) R_N_ and (B) G are significantly different following Ova challenge than their respective controls (n = 6−8/group) at 3 minutes while (B, C) G and H are markedly higher than controls at 20 minutes. (D) Illustration of normalized parameters for the Ova-challenged animals (*p<0.05, **p<0.01).

At 3 minutes after Ova challenge, R_N_ and G increased significantly compared to baseline (p<0.05, n = 8−10, figure 1 A, B). R_N_ declined subsequently so that by 20 minutes after challenge it was no longer significantly increased. However the elevation in G persisted (p<0.01, figure 1A, B). H was not increased at 3 minutes but was significantly increased at 20 minutes (p<0.05, figure 1C). Normalized parameters for the Ova-challenged animals illustrate the relative progression in the individual parameters over 20 minutes (figure 1D).

**Figure 2:**
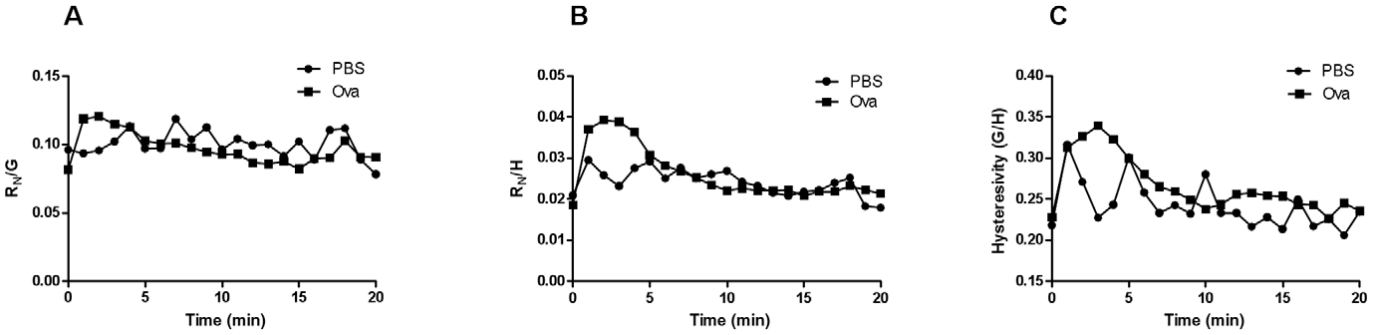
R_N_/G, R_N_/H and G/H over the 20 minute period following aerosol challenge with PBS or Ova. While there were no differences in (A) R_N_/G, (B) R_N_/H and (C) G/H were significantly elevated at 3 minutes after Ova challenge (n = 6−8/group). At 20 minutes there were no significant changes (*p<0.05).

At 3 minutes, there was no significant difference in R_N_/G compared to baseline suggesting that these parameters are coupled (figure 2A). However, R_N_/H was significantly higher than controls at 3 minutes (p<0.05, figure 2B). Similarly, G/H or hysteresivity, the ratio of energy dissipation to energy conservation, was higher at 3 minutes in Ova-challenged animals compared to controls (p<0.05, figure 2C) but not different amongst groups at 20 minutes.

### Airway narrowing: morphometric assessment

**Figure 3:**
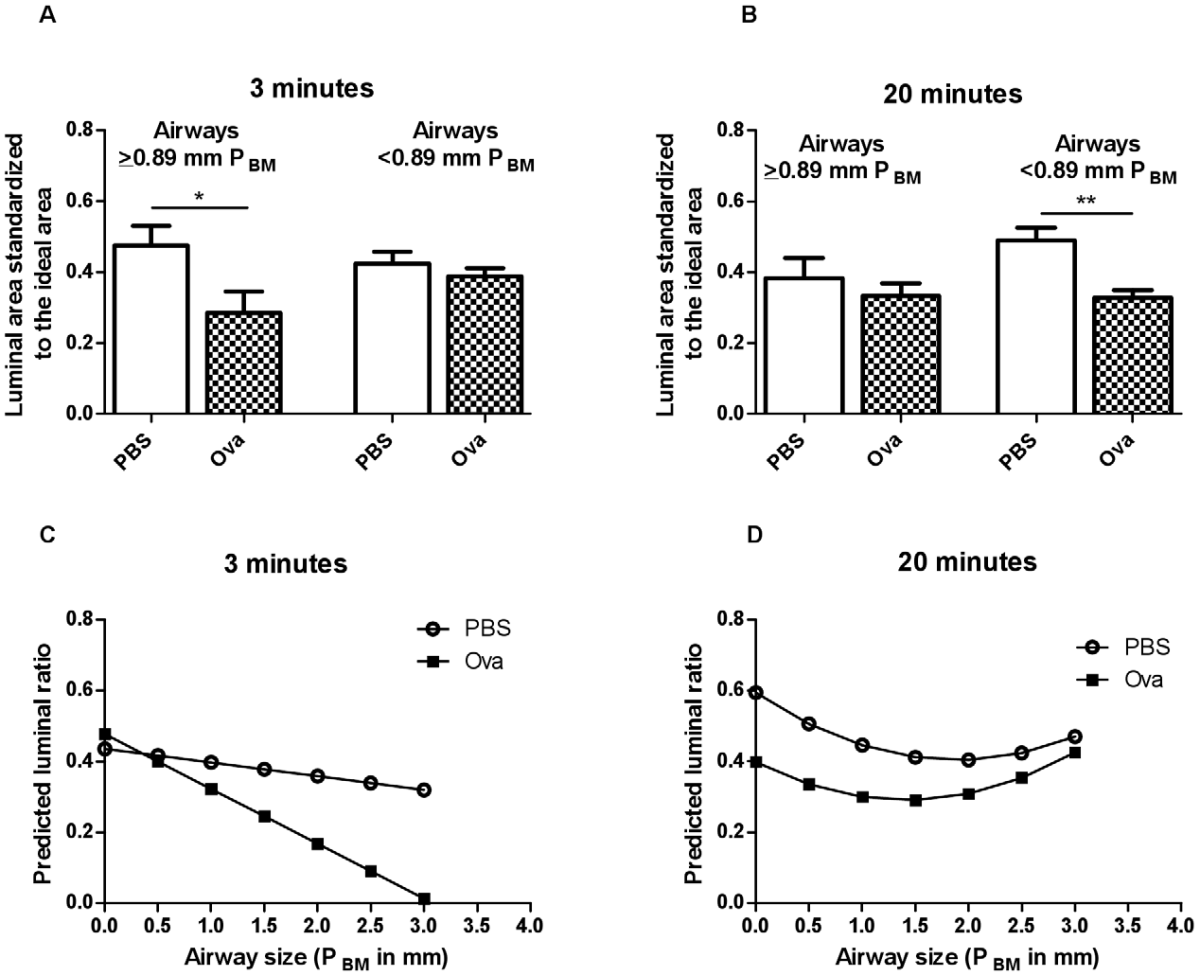
Airway Narrowing Morphometry at 3 and 20 minutes after aerosol challenge with PBS or Ova. Statistics were performed using the median airway size to assign airways >0.89 mm as medium/large airways and <0.89 mm as small airways.Comparison of morphometric data between the Ova and PBS-challenged animals was performed with a linear mixed model. (A) At 3 minutes, only larger airways in Ova compared to PBS were significantly different (C) 0.86 P_BM_ was the minimum cut-off to observe such differences. (B) At 20 minutes, only the smaller airways indicated significant differences between the treatment groups and (D) with increasing airway size, the luminal ratio difference between the treatment groups diminished. (n = 5−6, *p<0.05,**p<0.01).

Airway narrowing was assessed from the ratio of the lumen area to the ideal lumen, the area corresponding to the airway circumference calculated for a perfect circle. The median airway P_BM_ of all the airways assessed was 0.89 mm. Three minutes after Ova challenge, only the airways ≥0.89 mm were relatively more constricted than the comparable airways in controls (p<0.05, n = 5−6, figure 3A). At 20 minutes, the airway narrowing in the Ova-challenged animals was greater in the airways with a P_BM_<0.89 mm (p<0.01) and larger airways were no longer significantly narrowed (n = 5−6, figure 3B).

We tested the sensitivity of the statistical significance in differences in larger airway narrowing induced by Ova challenge to the choice of cut-off of airway size. The minimum cut-off which gave significance at 3 minutes was 0.86 mm, corresponding closely to the median airway size, 0.89 mm in P_BM_. At 3 minutes in the Ova group, the luminal ratio declined as airway size increased as did the difference in luminal ratio between the Ova and PBS groups (figure 3C). However, at 20 minutes, the difference in luminal ratio between Ova and PBS diminished as airway size increased (figure 3D). At >1.77 PBM, the luminal ratio difference between Ova and PBS groups was no longer significantly different.

### Heterogeneity of airway narrowing

**Figure 4:**
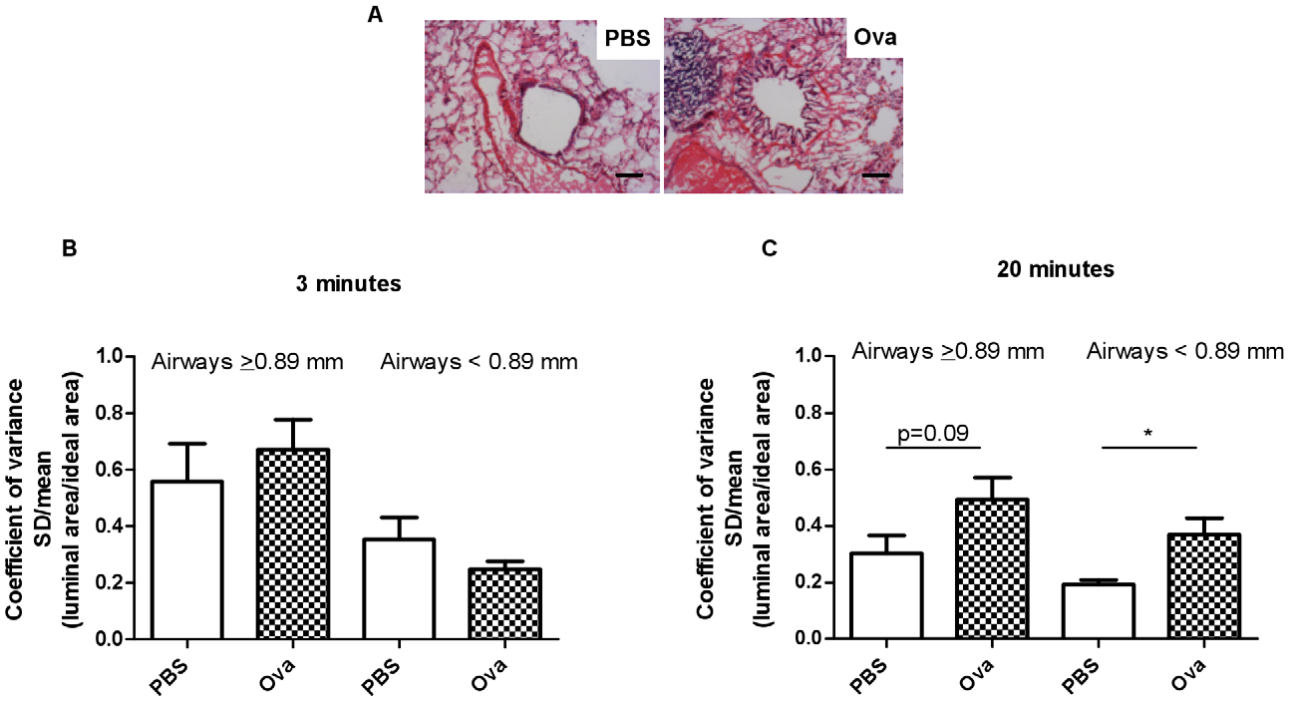
Airway narrowing heterogeneity at 3 min and 20 min after aerosol challenge with PBS or Ova. (A) Illustration of bronchoconstriction in PBS and Ova-challenged animals (Scaling bar = 100 µm). (B) While there were no differences to report at 3 minutes (n = 5−6), (C) airway narrowing heterogeneity was greater in the Ova-challenged animals compared to controls at 20 minutes (n = 4−6, *p<0.05).

Figure 4A is an illustration of hematoxylin and eosin staining in an airway from a PBS and Ova-challenged animal respectively. At 3 minutes after Ova or PBS challenges there were no significant differences in the coefficients of variation of luminal areas amongst groups (n = 4−6, figure 4B). In comparison at 20 minutes, the smaller airways (<0.89 mm) showed significantly greater heterogeneity of airway narrowing after Ova challenge compared to the controls (p<0.05, n = 6) while there was a trend (p = 0.09) for the airways with a P_BM_ ≥0.89 mm to also show heterogeneity of airway narrowing compared to their respective controls (figure 4C).

### Potential determinants of the distribution of airway narrowing during the EAR

We wished to explore some of the possible determinants of differences in the distribution of airway narrowing from large to small airways. There were no clear differences in the distribution of the mast cells in non-Ova-challenged animals between airways of P_BM_ ≥0.89 mm and <0.89 mm (figure S1A).

ASM mass in Ova-sensitized, non-challenged animals was normalized for the square of the P_BM_ to correct for airway size. There were no differences in the normalized SM as a function of airway size (figure S1B).

### Surfactant effects on the mechanical parameters and on airway narrowing as assessed by morphometry during the EAR

**Figure 5:**
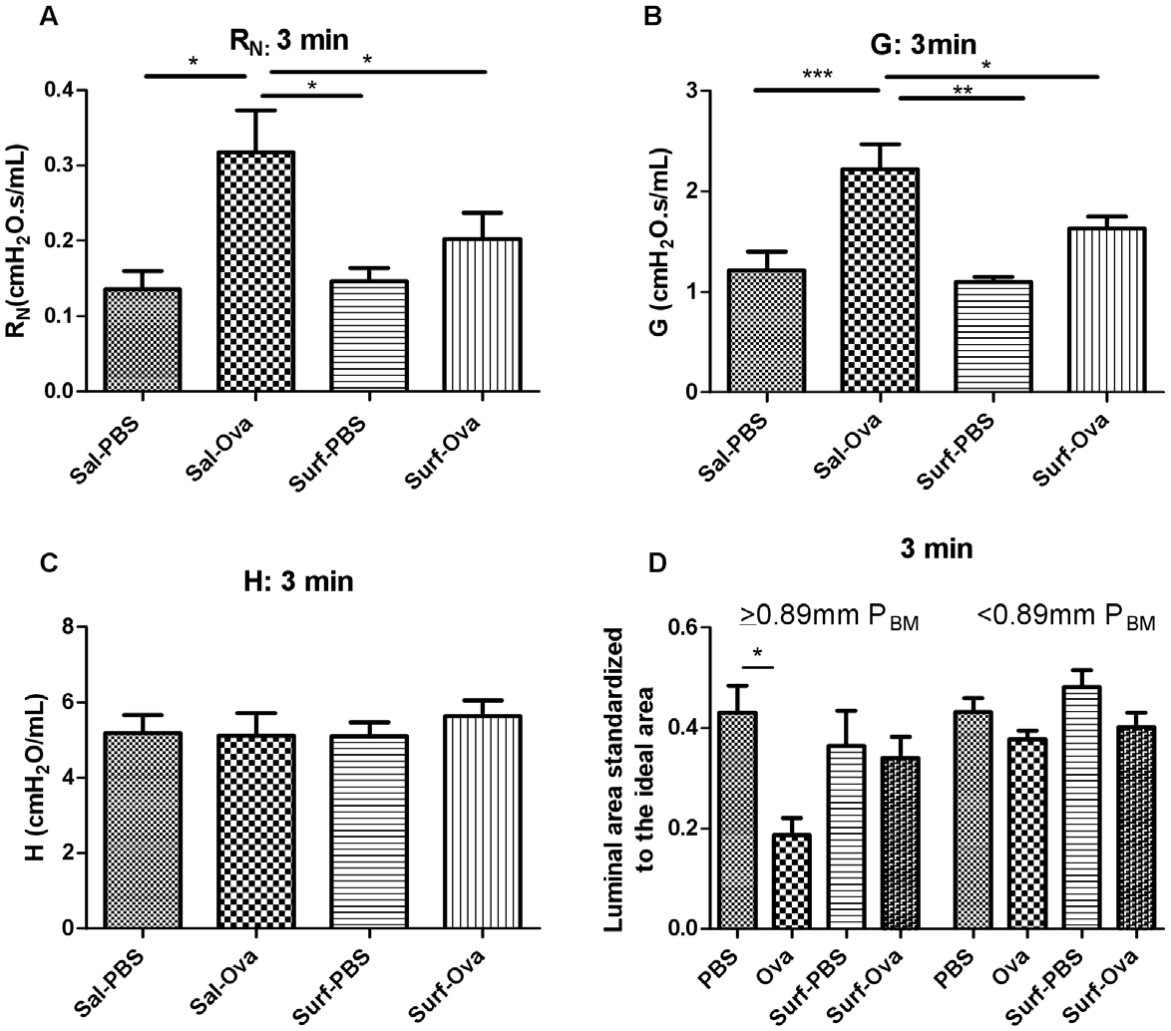
Surfactant effects on the constant phase parameters of AHR and corresponding morphometric assessments. The following groups were compared: PBS (n = 10), Ova (n = 11), Surf-PBS (n = 6), and Surf-Ova (n = 12). (A) R_N_ and (B) G at 3 minutes were inhibited by exogenous, natural surfactant administration, (C) H at 3 minutes, (D) The larger airways are bronchoconstricted as assessed by morphometry but the surfactant effects did not reach significance (n = 4−7, *p<0.05).

At 3 minutes after challenge there was a significant increase in R_N_ from baseline in Ova- challenged animals which was decreased by exogenous surfactant administration (p<0.05, figure 5A). The G parameter in the Ova-challenged animals increased significantly at 3 minutes (p<0.001, figure 5B) and the increase in G was prevented by pre-treatment with exogenous surfactant (p<0.05, figure 5B), while there were no changes in the H parameter (figure 5C). In both R_N_ and G, there was also a marked difference between the surfactant-administered, Ova-challenged and the saline administered, Ova-challenged groups (p<0.05, figure 5A and p<0.01 figure 5B respectively). By 20 minutes after Ova challenge R_N_, G and H parameters were not significantly different from baseline values (data not shown).

In the Ova-challenged animals at the peak of EAR, the airways >0.89 mm P_BM_ were significantly bronchoconstricted (p<0.05, figure 5D). The morphometric data for the airways >0.89 mm showed a lesser degree of airway narrowing after Ova challenge in animals pre-treated with surfactant. There were no significant differences in the Ova-challenged animals compared to controls in the airways <0.89 mm (figure 5D). Given the lack of changes in mechanical parameters at 20 minutes, morphometry was not performed at this time point.

### Effects of surfactant on inflammatory mediators (cysteinyl leukotrienes and amphiregulin) in the BAL fluid

**Figure 6:**
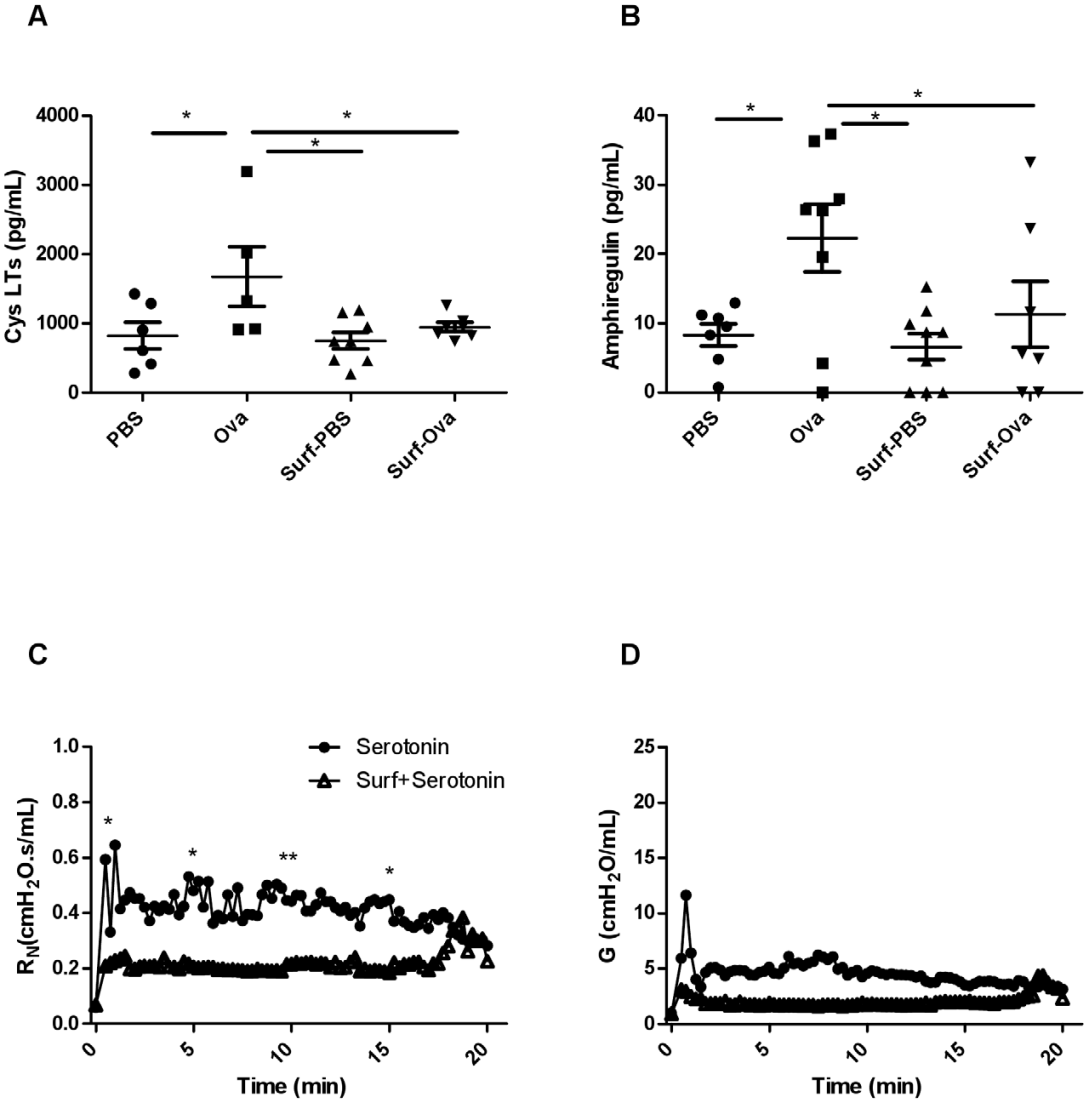
Mast cell–derived mediators in BAL fluid and in vivo assessment of EAR responses with serotonin-mediated bronchoconstriction in the presence of surfactant BAL was performed at 3 minutes after PBS or Ova challenge and mediators were measured by EIA or ELISA kits. (A) Cysteinyl leukotrienes (n = 5−8/group) and (B) amphiregulin levels (n = 7−9/group) (*p<0.05), (C) Serotonin-induced bronchoconstriction is inhibited by pre-treatment with surfactant, as observed in R_N_ at the peak response as well as at 5, 10, and 15 minutes after allergen challenge (n = 7, *p<0.05, **p<0.01). (D) At 5 minutes into the response, there is a trend for the inhibition of the G parameter by surfactant (p = 0.08) as well as at 10 minutes (p = 0.065).

Assessment of the concentration of total CysLTs from the BAL at the peak of the EAR in the study groups revealed an elevated concentration in the Ova-challenged animals compared to controls (p<0.05, n = 5−7, figure 6A). This increase in CysLTs was abrogated in animals that received exogenous administration of surfactant (p<0.05, n = 5−8, figure 6A). Similarly, surfactant prevented the increase in amphiregulin that was induced by Ova-exposure during peak EAR (p<0.05, n = 7−9, figure 6B).

### *In vivo* assessment of EAR responses with serotonin-mediated bronchoconstriction in the presence of surfactant

Serotonin was administered at a high dose to induce bronchoconstriction with a time course not dissimilar to the EAR. Following serotonin R_N_, G and H increased. Surfactant reduced the change in R_N_ at both the peak of the response, and at 5, 10 and 15 minutes into the response (n = 7, p<0.01, p<0.05, figure 6C). There was a trend for the reduction of G at 5 and 10 minutes (p = 0.08 and 0.065 respectively, figure 6D) while H was not significantly altered at any time point (data not shown).

### Effects of surfactant on RBL cell activation by Ova

**Figure 7:**
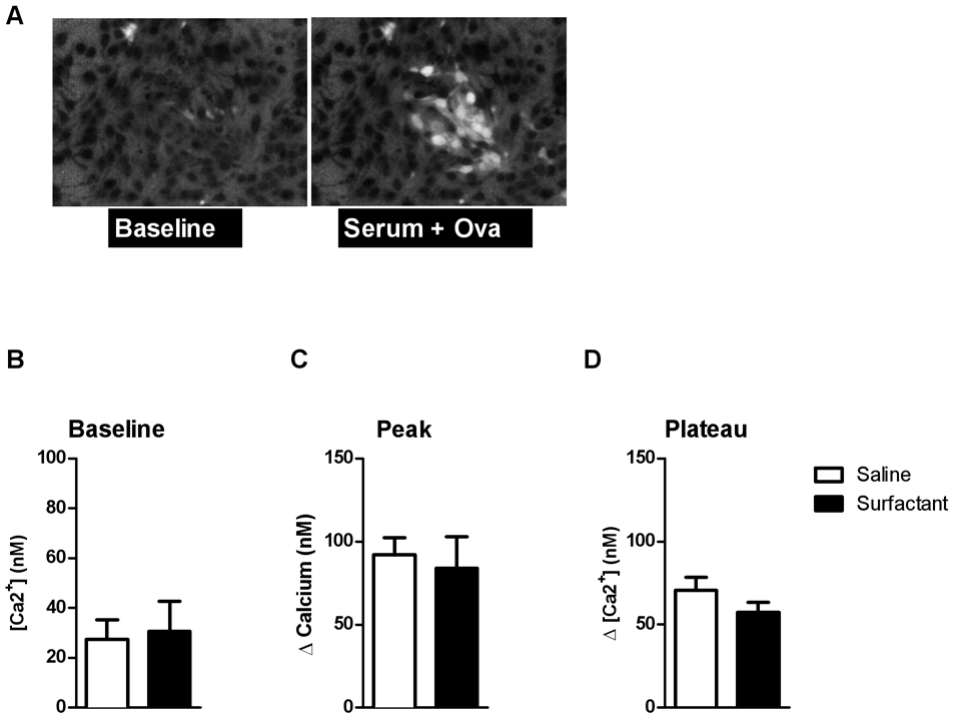
Effects of surfactant on RBL cell activation by Ova. (A) Images demonstrating Ca^2+^ release in mast cells, upon addition of serum and Ova. (B) There were no differences in the baseline values of the calcium with and without surfactant (n = 5−6). (C) The peak increase in calcium (n = 5−6) and the (D) subsequent plateau were not significantly attenuated by prior treatment with surfactant (n = 5−6).

RBL cells were used to model the effects of surfactant on Ova-mediated mast cell activation. First we tested the effects of surfactant on the detection of calcium in a cell-free FURA-2 solution. As illustrated in figure S2A for Ca^2+^ concentrations of 0 and 39 µM, we observed that the fluorescence of FURA-2 was altered with surfactant (n = 5−6, figure S2B) and we applied a correction to the calibration to account for the signals in the RBL cells in the presence of surfactant. Figure 7A illustrates mast cell responses upon addition of serum and Ova. There were no differences in the baseline intracellular calcium concentrations with and without surfactant (n = 5−6, figure 7B). Neither the peak nor the plateau response in calcium were significantly affected by the presence of surfactant (n = 5−6, figure 7 C,D).

## Discussion

Ova exposure triggered a significant EAR in sensitized animals as determined from changes in respiratory mechanical properties of the respiratory system. The peak of the EAR occurred at ∼3 minutes after the challenge at which time both of the parameters R_N_ and G of the constant phase model were elevated. By 20 minutes, R_N_ was no longer significantly elevated but G and H were. Narrowing of the large airways (>median size) assessed by morphometry was present at 3 minutes after challenge and had resolved by 20 minutes whereas the narrowing of the smaller airways (<median size) was not evident at 3 minutes but was present at 20 minutes. Surfactant administered prior to allergen challenge inhibited the changes in mechanical parameters reflecting large airway constriction at 3 minutes after challenge. Surfactant inhibited the release of the mast cell-derived inflammatory mediators, cysteinyl leukotrienes and amphiregulin, at the EAR peak. *Ex vivo*, surfactant failed to inhibit Ova-induced calcium release from RBL cells, indicating that the amelioration of airway narrowing by surfactant is more likely attributable to indirect effects on mast cells.

Following Ova challenge, R_N_ and G rose quickly and synchronously whereas the changes in H lagged in time. The changes in R_N_ were transient whereas changes in G persisted over the 20 minute period of observation. R_N_/G was not significantly elevated at peak EAR suggesting that these parameters may share common determinants, namely narrowing of the airways larger than the median value or that they may reflect simultaneous effects on different regions within the airway tree and lung parenchyma. The change in R_N_ is usually attributed to airway resistance and G to tissue damping [1], [23]. The elevation in G at a time when R_N_ had partially recovered is consistent with the idea that the factors contributing to R_N_ and G are not identical. In contrast, R_N_/H and G/H were significantly different at peak EAR, also suggesting that H is determined by changes in the peripheral lung tissues and beyond the airways captured by changes in R_N_ and G. In order to assess the contributions of the narrowing of airways of different sizes to the parameters of the constant phase model, we snap-froze lungs following allergen challenge at a transpulmonary pressure that fixed lung volume in the tidal breathing range, using previously published methodology [22]. We divided airways into two categories, larger and smaller than the median value (0.89 mm internal perimeter) in order to relate morphometric measurements of airway narrowing to the constant phase parameters. The morphometric data showed narrowing of the larger airways at the peak EAR and recovery by 20 minutes. The converse was observed for smaller airways that were narrowed significantly at 20 minutes but not at 3 minutes after challenge. These data are in concordance with the interpretation offered for the parameters of the constant phase model, although it does appear that the G parameter is sensitive to both large and small airway narrowing.

The heterogeneity of airway narrowing seems likely to be dependent on the fact that airway narrowing was induced by aerosol challenge [22] although a substantial contribution from local mechanisms and not solely non-uniform agonist delivery has also been suggested [24]. There were no differences in heterogeneity of airway narrowing at 3 minutes after challenge amongst any of the groups. By 20 minutes after Ova challenge, the heterogeneity of airway narrowing markedly increased in the smaller airways and showed a trend for increase in the larger airways. The coefficient of variation of the airway luminal sizes in the PBS-challenged group was higher at 3 minutes than at 20 minutes suggesting that there was a response in the larger airways to PBS, albeit resulting in a much smaller observable effect on respiratory system mechanics. The heterogeneity of constricted airways may be analyzed via direct visualization by morphometric analysis on frozen lungs [25], by hyperpolarized 3-helium dynamic MRI [26], high-resolution computerized tomography [27]–[29], synchrotron radiation computerized tomography [30], mechanical measurements such as the alveolar capsule technique [31] and by forced oscillation and model fitting to whole lung impedance data [32], [33]. The heterogeneity of small airway narrowing observed for Ova-challenged animals correlated with the G and H parameters.

We explored several of the important potential determinants of the site and magnitude of Ova- induced airway narrowing such as mast cell density and ASM mass. We found no clear differences in mast cell distribution across airways of increasing size and no significant airway size dependence of SM mass. There are several factors which should favour a greater narrowing of the smaller airways for an equivalent allergen challenge. Firstly, the decrease in airway radius should reduce the wall tension required to create a given transmural pressure, based on the Laplace relationship [34]. For similar reasons, surface tension within the small airways should favour airway narrowing ([35] and figure S3) and the effect would be expected to be markedly enhanced by the disruption of surfactant properties by the microvascular leak of protein that has been shown to occur during the early allergic response [9]. We speculate that, as been previously suggested, the more sustained abnormality in peripheral lung mechanics may be attributable to airway closure [36], favored by high surface tension and the development of fluid menisci within some of the small airways. The surface tension of the fluid in the lungs *in vivo* has been estimated to be between 5 and 30 dynes/cm or mN/m [37]. Forty-eight hours after antigen challenge, there is reportedly a dysfunction in pulmonary surfactant [38]. It was later reported that in asthmatic humans, phospholipid alteration in surfactant, particularly in phosphatidylglycerol, occurs after antigen challenge [12]. Furthermore, in stable asthmatic individuals, dipalmitoyl phosphatidylcholine, the primary phospholipid found in surfactant is reduced in sputum but not BAL surfactant [39]. Small airways are most at risk of closure and an intervention to decrease surface tension may decrease the risk of airway closure. In small animals the distribution of the surfactant-producing Clara cells throughout the airways suggests that surfactant may have such a role even in larger sized airways in small animals [40]. In order to explore the plausibility of disruption of surfactant function within the airways by protein leakage we calculated the transmural pressure that might be expected as a result of surface tension as a function of airway size using the Laplace relation (figure S3). Based on our experimental data, surfactant had protective effects in inhibiting airway obstruction. We had postulated this effect would be most evident in the peripheral airways as this is the site where surfactant is endogenously more abundant and has the greatest effect on transmural pressure. However, the inhibition of the peak EAR, as well as the reduction of bronchoconstriction of the larger airways, indicated that surfactant was protective in larger airways. The aerosolization of saline for 5 minutes prior to Ova challenge also appears to have modified airway responses, as the G and H parameters in these animals at 20 minutes post Ova challenge were not statistically significantly different from baseline values, in contrast to the animals that did not receive any aerosolization prior to allergen challenge.

Synthetic surfactant has been described to inhibit early allergic airway responses in human subjects, presumably related to effects on surface tension. Collectins have been associated with the inhibition of various mediators including the IgE-mediated degranulation of mast cells [41]. However surfactant proteins A and D are not present in the surfactant preparation that we used. A protective role of inhaled synthetic, protein-free, phospholipid-based surfactant in the early allergen-induced response in humans has also been described [42]. To address the possibility that the surfactant we employed, lacking immunomodulatory proteins, might have a role in modulating inflammatory mediators, we assessed the concentrations of CysLTs in the BAL in BN rats immediately after the EAR peak was reached. CysLTs were significantly inhibited by exogenous surfactant administration. We observed similar results with amphiregulin. Amphiregulin, an epidermal growth factor receptor ligand, is reported to be present in 70% of the mast cells from human asthmatic airways and human cord blood-derived mast cells (CBMCs) secrete amphiregulin upon IgE crosslinking [43]. It is released by epithelial and mast cells and could potentially be important in the EAR as it has been reported to be elevated after an acute asthma attack in humans [44].

We examined the effects of this surfactant preparation on the activation of RBL-2H3 cells, a frequently used model for the mast cell. The effects of surfactant appear to be indirect on the mast cells since activation by Ova and sensitized serum *ex vivo* was not reduced by treatment with surfactant. However bronchoconstriction by mechanical effects *per se* does not lead to release of mast cell mediators [45] and it is therefore not clear how a reduction in surface tension *per se* may have reduced mediator release. Surfactant also reduced the serotonin-induced large airway narrowing, indicating that the effects of mediators released by mast cells were also likely attenuated.

In summary, ovalbumin exposure induced a significant EAR, in which the peak of the response occurred as early as ∼3 minutes after the challenge. Mechanical assessments and morphometry on frozen tissues indicated that the narrowing of the large airways occurred transiently and resolved quickly whereas the peripheral airway narrowing was more persistent. Changes in R_N_ appear to reflect narrowing of airways greater than the median-sized airway whereas G is affected by airway narrowing in airways that are larger or smaller than the median-sized airway. Exogenous surfactant administration decreased the peak R_N_ and G and morphometric large airway narrowing in Ova-challenged animals, attributable to inhibition of mast cell mediator release and attenuation of the bronchoconstriction induced by mediators that are released.

## Supporting Information

Figure S1 **Possible structural determinants of the site of the early airway response; mast cells, ASM in Ova-sensitized but unchallenged animals.** (A) There was no relationship between airway size and the density of mast cells (n = 8). (B) The size corrected area of ASM was not different in larger (>0.89 mm P_BM_) compared to smaller airways (<0.89 mm P_BM_) (n = 6).
(TIF)

Figure S2 **Effects of surfactant on RBL cell activation by Ova.** (A) Examples of responses at 0 and 39 µM, respectively, in a cell-free mix. (B) The fluorescence of FURA-2 was altered with surfactant (n = 5−6, *p<0.05).
(TIF)

Figure S3 **Possible determinants of early airway response; surface tension.** We calculated the transmural pressure that might be required to overcome surface tension as a function of airway size using the Laplace relation: P = 0.0102•γ/r where P is transmural pressure in cmH_2_0, γ is surface tension (ST) in dynes/cm, r is airway radius in mm and the factor of 0.0102 converts from Pa to cmH_2_0. The vertical line is indicative of ST contributing to transmural pressure at the mean P_BM_ (0.89 mm). The ST of water is 70 dynes/cm while the ST of fluid in the lining of the lung is between 5–30 dynes/cm.
(TIF)
