# Supplementary figures and images for: Site of Allergic Airway Narrowing and the Influence of Exogenous Surfactant in the Brown Norway Rat
Source: PLoS One. 2012 Jan 19;7(1):e29381. doi: 10.1371/journal.pone.0029381 (PMC3261862; doi:10.1371/journal.pone.0029381)

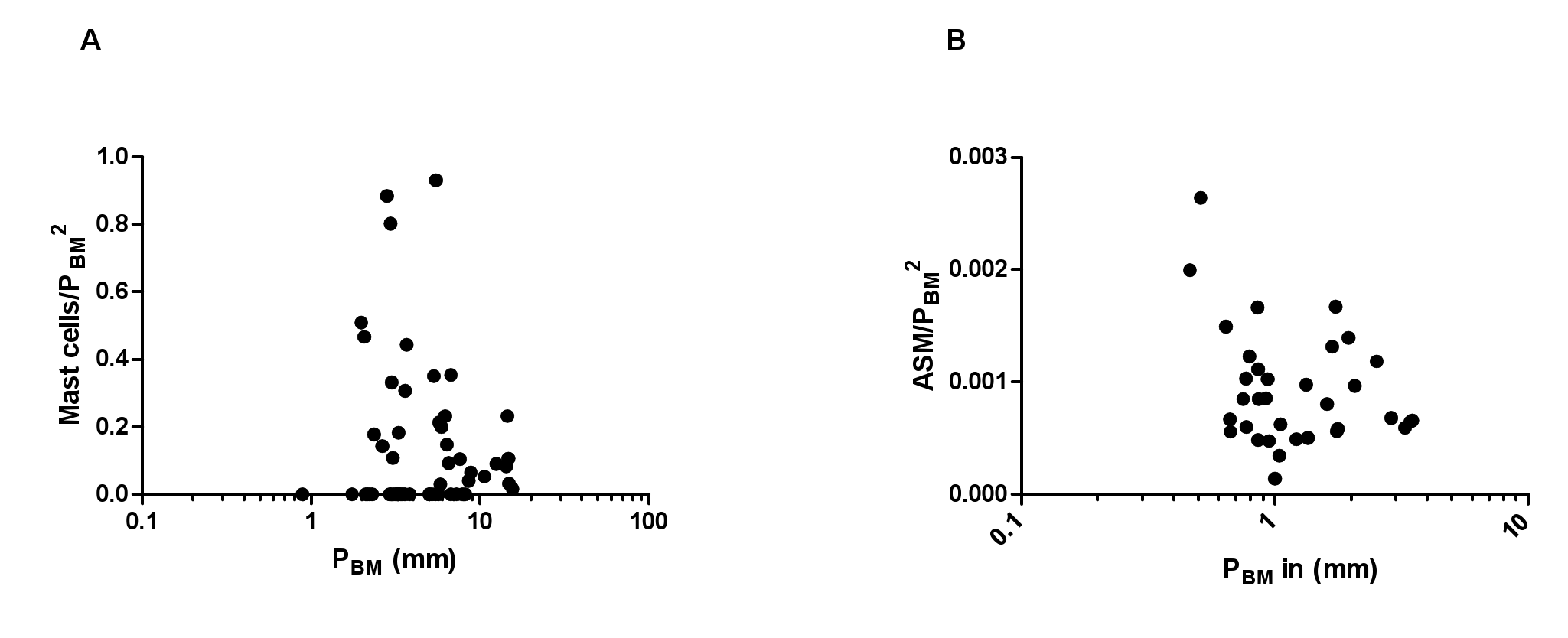

Supplement: Figure S1 — Possible structural determinants of the site of the early airway response; mast cells, ASM in Ova-sensitized but unchallenged animals. (A) There was no relationship between airway size and the density of mast cells (n = 8). (B) The size corrected area of ASM was not different in larger (>0.89 mm PBM) compared to smaller airways (<0.89 mm PBM) (n = 6). (TIF) [file pone.0029381.s001.tif]

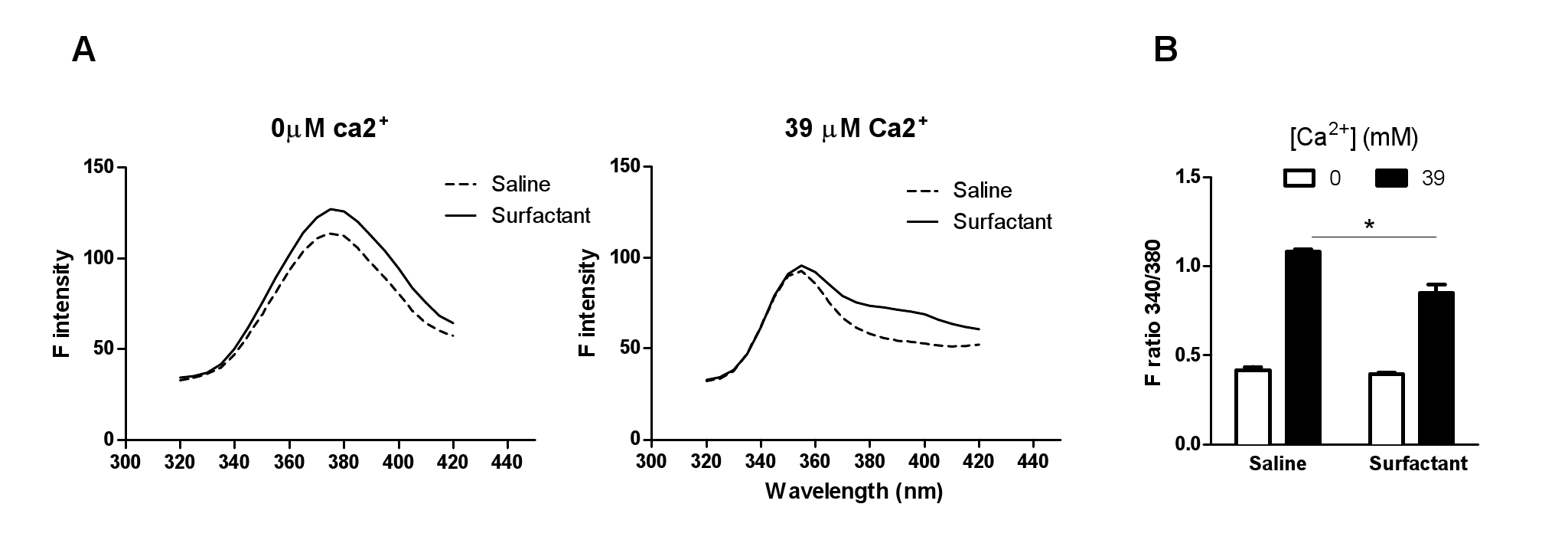

Supplement: Figure S2 — Effects of surfactant on RBL cell activation by Ova. (A) Examples of responses at 0 and 39 µM, respectively, in a cell-free mix. (B) The fluorescence of FURA-2 was altered with surfactant (n = 5−6, *p<0.05). (TIF) [file pone.0029381.s002.tif]

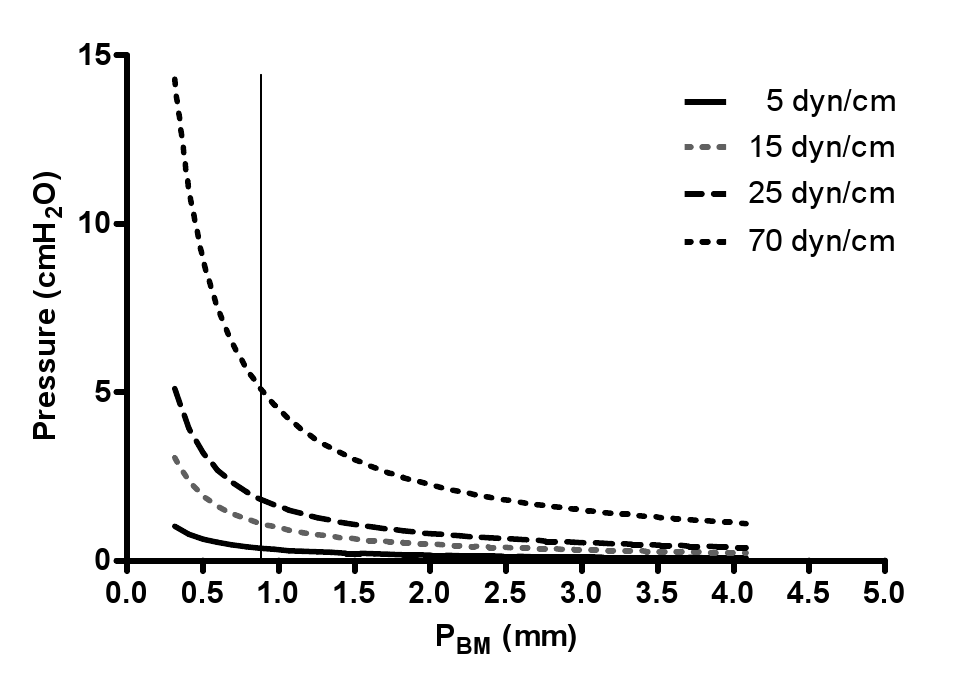

Supplement: Figure S3 — Possible determinants of early airway response; surface tension. We calculated the transmural pressure that might be required to overcome surface tension as a function of airway size using the Laplace relation: P = 0.0102•γ/r where P is transmural pressure in cmH20, γ is surface tension (ST) in dynes/cm, r is airway radius in mm and the factor of 0.0102 converts from Pa to cmH20. The vertical line is indicative of ST contributing to transmural pressure at the mean PBM (0.89 mm). The ST of water is 70 dynes/cm while the ST of fluid in the lining of the lung is between 5–30 dynes/cm. (TIF) [file pone.0029381.s003.tif]
